# Father Involvement in the Lives of Their Children With Intellectual and Developmental Disabilities in the UK

**DOI:** 10.1111/jar.70091

**Published:** 2025-07-05

**Authors:** Emma Langley

**Affiliations:** ^1^ Education Studies University of Warwick Coventry UK

**Keywords:** developmental disabilities, fathers, intellectual disabilities, involvement, parenting

## Abstract

**Background:**

Father involvement in parenting has received scarce attention in the disability field. This qualitative study explored the involvement, roles and responsibilities of fathers of individuals with Intellectual and Developmental Disabilities (aged 5–24 years).

**Methods:**

Thirteen fathers participated in online, semi‐structured interviews. Fathers were asked to describe their involvement in caregiving, their roles and responsibilities, and the factors that had an impact on these domains.

**Results:**

Fathers were involved in the direct and indirect care of their child, and provided support for their partner and any siblings, alongside work commitments. A range of individual, interpersonal and contextual factors impacted their involvement and parenting role.

**Conclusions:**

Fathers play an important role in the lives of children with Intellectual and Developmental Disabilities. Ways in which services could improve the quantity and quality of paternal involvement in parenting are discussed.

**Summary**

- Fathers can be involved in the direct and indirect care of their child with Intellectual and Developmental Disabilities; however, there are a range of factors that can act as a barrier to involvement, which need to be attended to.
- Ways in which professionals working with families of individuals with Intellectual and Developmental Disabilities can better support fathers and their children have been identified.
- The study contributes new knowledge on the involvement of fathers of individuals with Intellectual and Developmental Disabilities of different ages and has considered how existing mainstream models of paternal involvement could be applied within the context of disability.

## 1 Introduction

In Western societies, men are more involved in the care of their children than they have ever been (Faircloth 2014), and are more likely than their own fathers to take on parenting roles and responsibilities (Gough 2014). The roles and responsibilities of fathers of individuals with Intellectual and Developmental Disabilities have received scarce attention in the literature (Blacher et al. 2020), with the majority of the existing research focused on risk to paternal mental health (Dunn et al. 2019; Giallo et al. 2015; Langley et al. 2020; Seymour et al. 2017). Some existing studies have reported that fathers are active in raising their child (i.e., sharing parenting duties with their partner) and want to be involved in making decisions about their child; however, it is often their partner/child's mother who takes the lead due to being the primary caregiver, and so fathers often take a supporting role (Rafferty et al. 2020). Role specialisation has also been reported in families of autistic children and adolescents, with fathers more likely to be in paid employment and less involved in childcare (Hartley et al. 2014).

Despite individuals with Intellectual and Developmental Disabilities being more likely to live with their parents in adulthood, most studies in this field are conducted on parents of school‐aged children. Existing cross‐sectional studies with fathers of older children (20–30 years old) have found that fathers report wearing different ‘hats’, such as a parent and breadwinner (Dunn et al. 2021), and take on multiple roles (caregiver, supervisor and educator) at the same time (Schippers et al. 2020). Fathers describe needing to spend more time caring for their child due to increased need, and say it is their job to guide their children and teach them the ‘rules of life’ (Schippers et al. 2020). Fathers of older children have also noted their preference to be called a parent over ‘carer’ as the label of carer was deemed impersonal and ignored the fact that fathers were still fathers, even if their child was an adult and needed care (Dunn et al. 2021; Thackeray and Eatough 2018). To date, there has been one longitudinal study on fathers of children with Intellectual and Developmental Disabilities that captured Swedish fathers' experiences of parenting over a 5‐year period (Boström and Broberg 2014). They describe the initial adjustment of fathers as they come to terms with their child's diagnosis, and that the level of involvement and engagement of fathers varied extensively between fathers and over time. It is clear that more work needs to be done to capture the perspectives of a broader age range of fathers in relation to their role and involvement, and the kinds of factors that may impact this.

There is no overarching theory related to fathers of disabled people (Blacher et al. 2020), therefore we look to other theoretical models in the mainstream parenting literature. The ecological model of father–child relationships (Cabrera et al. 2014) contextualises fatherhood, exploring a range of individual (father characteristics, father child‐rearing history, child characteristics and development) interpersonal (parenting behaviours, family relationships) and contextual (work, social networks and community, socioeconomic status) factors that can have an impact on the bidirectional relationship between a father and their children. All aspects of this model have the potential to be influenced by fathering a disabled child; however, the heterogeneity of Intellectual and Developmental Disabilities means that fathers could have very different experiences and levels of involvement in caregiving, depending on the type and severity of their child's needs.

Other theories, such as that by Pleck (2012), state that when thinking about father involvement, we should not just be thinking about how much a father is involved in their child's life (i.e., the quantity of time they spend with their children), but what fathers *do* with their children (the quality of those interactions). This is pertinent given the additional demands of parenting a disabled person over their life course, and is inclusive of fathers who may not be with their child all of the time. Pleck (2012) also describes how father involvement can be separated into different domains, such as direct and indirect care. Direct care involves father–child interaction in the form of daily activities, such as caregiving, play and education. Whereas indirect care is about arranging and taking responsibility for things related to their child, for example, arranging care, resources and services for a child. Both of these domains of involvement are relevant to fathers of children with Intellectual and Developmental Disabilities, in particular, the additional and long‐term indirect care demands. To date, these aspects of paternal involvement have received little attention within the field.

The current study aims to address the lack of research on the roles and responsibilities of fathers of children, adolescents and adults with Intellectual and Developmental Disabilities. It was designed in response to a special issue on fathers of children with Intellectual Disabilities, which called for more research on fathers' direct role in caregiving and involvement (Blacher et al. 2020). Previous consultation by the author with a group of fathers of disabled children in 2019, also concluded that more studies were needed to draw attention to what fathers *are* doing with and for their families, and recognise that fathers at different stages of their parenting journey may have different experiences and wisdom to share.

The research posed two questions:

*How do fathers of children with Intellectual and Developmental Disabilities describe their involvement in caregiving and their roles and responsibilities?*

*What factors have an impact on the involvement, roles and responsibilities of fathers of children with Intellectual and Developmental Disabilities?*

## 2 Methods

### 2.1 Design

This was an exploratory study which aimed to describe the involvement, roles and responsibilities of fathers of children with Intellectual and Developmental disabilities. Semi‐structured interviews were conducted because of their ability to capture the in‐depth experiences of fathers and allow for flexibility in relation to the themes discussed.

### 2.2 Participants

Fathers were eligible for the study if they were: aged 18+, a biological, adoptive or step father of a child with intellectual and developmental disability, in regular contact with their child (but did not have to live in the same household), lived in the United Kingdom and had spoken English at a level to take part in an interview. Participants were purposely sampled to ensure that fathers of children, adolescents and adults were included in the study. The final sample included 13 fathers, 4 of children (aged 4–11 years), 5 of adolescents (12–17 years) and 4 adults (18+ years) (Table 1). Fathers were aged between 40 and 59 years. Most fathers were White British and were educated to degree level or above. With the exception of one stepfather, all were biological fathers. Two fathers had separated from their child's mother and lived with their child for part of the time. One father was widowed and lived with his children full‐time, and one adult child had recently transitioned into full‐time residential care. At the time of the research, the children were aged between 5 and 24 years (mean = 13 years). The majority were male and had a diagnosis of autism and intellectual disability (father‐reported), and had one sibling. Monthly household income ranged from £25,000–£149,000, with most fathers reporting that they (and their partner, where relevant) were ‘doing alright’ financially.

**TABLE 1:** Father demographic information and characteristics of their child with Intellectual and Developmental Disabilities.

Father pseudonym	Age of father	Ethnic background	Highest level of education	Living status	Total household income	Subjective poverty	Child age	Child gender	Child diagnoses	Number of siblings
Richard	50–59	Any other White background	Degree or graduate education	Child in residential care	£35,000–£34,999	Doing alright	18	Male	Autism	1
Jak	40–49	Asian or Asian British—Pakistani	Degree or graduate education	Living with child	£25,000–£34,999	Living comfortably	12	Male	Autism, Intellectual Disability	1
David	50–59	White—English	GCSE level education or equivalent	Living with child	Less than £25,000	Doing alright	18	Female	Profound Intellectual Disability	1
Sanil	40–49	Asian or Asian British—Indian	Post‐graduate education	Living with child	£150,000 or more	Living comfortably	11	Male	Autism, Intellectual Disability	1
Ranveer	40–49	Asian or Asian British—Indian	Degree or graduate education	Living with child	£75,000–£99,999	Doing alright	8	Female	Down Syndrome, Intellectual Disability	2
Callum	40–49	White—English	GCSE Level education or equivalent	Not living with child full‐time	£50,000–£74,999	Just about getting by	5	Male	Autism, Intellectual Disability	0
Peter	40–49	White—English	Degree or graduate education	Living with child	£50,000–£74,999	Doing alright	8	Male	Autism, Intellectual Disability	1
William	40–49	White—English	Post‐graduate education	Living with child	£50,000–£74,999	Doing alright	12	Male	Cerebral Palsy, Dyspraxia, speech delay	1
Alistair	50–59	White—Scottish	Vocational education	Not living with child full‐time	£35,000–£49,999	Just about getting by	19	Female	Autism	1
Martin	40–49	White—English	Degree or graduate education	Living with child	£35,000–£49,999	Just about getting by	12	Male	Autism, Intellectual Disability	2
Sidney	50–59	Asian or Asian British—Indian	Undergraduate education (e.g., University examinations but not completed degree)	Living with child	£50,000—£74,999	Doing alright	16	Male	Cerebral Palsy	0
Greg	50–59	White—English	Vocational education	Living with child	£35,000—£49,999	Just about getting by	24	Female	Autism, Intellectual Disability, Dyspraxia	0
Sean	40–49	White—English	Degree or Graduate education	Living with child	£100,000–£149,999	Living comfortably	16	Male	Autism	2

### 2.3 Procedure

At project inception, an advisory group including three fathers of children with Intellectual and Developmental Disabilities was formed. The father advisors were tasked to review participant recruitment materials (advert, participant information leaflet and consent forms) and the interview schedule, to ensure that the questions were appropriate for fathers (Yaremych and Persky 2023). The fathers were compensated for their role as ‘experts by experience’ (NIHR 2022).

The semi‐structured interview schedule was designed to gently steer the conversation to the involvement, roles and responsibilities of fathers. Given that there were no existing theoretical models of father involvement specific to those raising children with disabilities to draw upon (Blacher et al. 2020), theory outside of the disability field was explored when conceptualising the study and developing the interview schedule. The work of Pleck on father involvement in the general population (Pleck 2012) was particularly relevant as it focuses on the quality of paternal involvement and includes aspects of indirect care, which can be especially time‐intensive for parents of disabled children. Example questions in the interview schedule included: ‘What does a typical day with your child look like?’ and ‘What kinds of things are you responsible for in terms of your child's life?’ Fathers of adolescents and adults were asked to reflect on how these roles and responsibilities had changed over time. We then asked fathers to reflect on any factors that had influenced or impacted their involvement, roles and responsibilities. Prompts were guided by the ecological model of father–child relationships (Cabrera et al. 2014) and a review of the empirical literature in the field. Once the schedule had been designed by the research team it was then shared with the father advisors who reviewed the themes and questions and provided feedback, which was acted upon.

Full ethical approval was granted by the University Humanities and Social Sciences Research Ethics Committee in November 2021 (HSSREC 37/21‐22). A study webpage was created to include project information and host a short information video about what participation in the study would involve. This approach has been found to be successful when conducting interviews online or over the telephone, as it can supplement written information and allow participants to meet the researcher prior to the interview (Ladlow et al. 2021). Calls for participation were made on social media, and information was sent out via email to third‐sector organisations and existing networks of the author. Potential participants were directed to the study webpage, where they could view the study information and register their interest. Fathers who expressed an interest in the study were screened to ensure that they met the eligibility criteria and then an interview time was scheduled. All participants provided written informed consent and were given the opportunity to ask questions prior to the interview.

Individual interviews were conducted by the author and their research assistant between March and May 2022. Interviews were either conducted online or over the telephone, at a time that was convenient for the father, which tended to be during the evening. If the interview was conducted online, fathers were given the option to have their camera on or off. Where possible, researchers kept their cameras on to maintain a sense of connection. On average, interviews were 75 min in duration (range: 39–104 min). Interviews were audio‐recorded using a dictaphone, and researchers made their own notes and reflections during and after the interview. The recordings were then transcribed by a university‐approved transcription company. After the interview, participants received a £25 electronic Amazon gift voucher for their participation (NIHR 2022).

### 2.4 Analysis

The purpose of the study was to describe what father involvement in caregiving looked like and identify any factors that had an impact on their involvement. The thematic analysis (Braun and Clarke 2022) was therefore guided by this focus. Existing theoretical frameworks on father involvement in the general population (Cabrera et al. 2014; Pleck 2012) also guided the interpretation and presentation of the data.

In the first stage of the analysis, the author listened back to all of the recordings and revisited their notes made during and after each interview to re‐familiarise themselves with the data. Next, the transcripts were read separately by the author and the research assistant who applied codes (and descriptive‐level labels) to any data that they interpreted as relevant to the research questions. These codes were then reviewed by the author and consolidated into themes, which were assigned a name. For RQ1, it became apparent that fathers described their roles and responsibilities not only in relation to their disabled child, but also their partner and wider family; therefore, superordinate and subordinate themes were created to reflect this. For RQ2, themes influencing the roles and responsibilities of fathers were also grouped non‐hierarchically, according to whether they were individual, interpersonal, or contextual factors, guided by the ecological model of father–child relationships (Cabrera et al. 2014). Care was taken in the write‐up stage to describe the experiences of all the fathers, highlighting commonalities as well as alternative cases. Rich description has been supported by pseudonymised extracts from the data.

### 2.5 Researcher Positionality

The author considered and interrogated their own positionality throughout the study design, data collection, and analysis process. The deliberate inclusion of fathers with lived experience in the advisory group was in response to the author's position as an outsider to the disabled community and aimed to ensure that all aspects of the project were aligned with the interests of fathers. Ongoing written reflections about the data collection were recorded by the author (Sibbald et al. 2024) and revisited throughout the process of analysis and write‐up of the data. While the final themes were decided upon by the author, these were shared with fathers in the advisory group who provided a forum for discussion and challenge.

## 3 Results

### 3.1 RQ1: Father Involvement, Roles and Responsibilities

While there were natural variations in the amount of time that each father spent with their child (i.e., quantity), all described multiple roles and responsibilities that they performed for their disabled child and, where applicable, other family members. These have been organised under three superordinate themes (see Table 2).

**TABLE 2:** The involvement, roles and responsibilities of fathers of children with Intellectual and Developmental Disabilities.

Superordinate themes	Subordinate themes
Supporting their child (direct care)	Personal care, play and leisure activities
Supporting their child (indirect care)	Liaising with professionals, developing their own knowledge
Supporting their family^a^	Supporting their partner, protecting other children, providing for their family

^a^ In cases where this was relevant.

### 3.2 Superordinate Theme: Supporting Their Child (Direct Care)

#### 3.2.1 Subordinate Theme: Personal Care

A significant part of direct care included helping their child with their everyday personal care routines. For many fathers in two‐parent households, they classed their partner as the main caregiver; however, outside of work hours, they would try to share the load, for example, by getting their child ready in the mornings and evenings.

If I think about in the morning, it's getting her up, you know, changing her pullup and getting her to brush her teeth and then I always make—she loves scrambled egg in the morning, so I'll make her scrambled egg in the morning. So she eats well. You know, once I've done that and she's eaten, I get her changed, I get her dressed, and, yeah, make sure she's ready so when the school bus comes to collect her that she's ready to walk out the door and get on the bus and go really. And then in the evening, bathing her and getting her ready for bed and just drying her hair, which I love to do, I love to sit there and just dry her hair and she loves it as well, it's kind of therapeutic on both sides… (Ranveer)

Fathers of older children talked about extensive involvement in the personal care of their child into adulthood and how care was becoming more challenging as their children matured physically and emotionally:

She's got more forceful, bigger, more hard to deal with, more emotional, hormones, the change the pads and clearing up and blood and stuff so you know it's the sort of thing. Most dads they don't have to deal with [this]… (Greg)

For fathers of autistic children, there was an emphasis on maintaining routines and rituals, which helped their child (of any age) feel settled. One father, Martin, talked about the importance of saying and doing the right things, while another talked about how he loved reading the same book to his son every night:

I was reading a book to him and it's the 100th time that we've read this book. It's about counting. So, you count one acorn, two apples…then one page is a hundred stars and I will count every single star every single night because he wants it. (Callum)

One father spoke about the recent, painful decision of letting his adult son go into full‐time supportive living as they could no longer cope with his needs at home. He said that his role as a father was the same as that of a role of a mother—to try and support their child. Not being able to care for their son at home was incredibly painful and had an impact on how he felt about himself:

There are many days when I just think I'm useless because my son is in care…not knowing what your own son is feeling, not being able to help him, not being able to look after your own child, having had to admit that you can't cope with your own child would I think hurt anybody. (Richard)

#### 3.2.2 Subordinate Theme: Play and Leisure Activities

The accounts of fathers reflected aspects of their role that brought joy to them and their child. This often centred around play for fathers of young children and leisure activities for fathers of adolescents and adults. While for some, the type of activities they now do with their child was different to what they had imagined when their child was born, many described engaging in activities that their child enjoyed and the close relationship they now had with their child:

[He is] quite loving and caring and he's very funny, got really good sense of humour, he's quite physical and tactile, he likes like hugging and kissing and squeezing…which is great, you know, because you're not… when you think about ASD, some children might really struggle with that, but I'd say that's the opposite. (Jak)

I'll do a bit of wresting with him and sometimes, I'll just sit with him if he's calm. And other times, we'll sing songs and dance and we'll have a bit of a play. (Peter)

### 3.3 Superordinate Theme: Supporting Their Child (Indirect Care)

#### 3.3.1 Subordinate Theme: Liaising With Professionals

Overall fathers were less involved in indirect care compared to their partner/child's mother. However, when it came to arranging care for their child and liaising with professionals, some fathers took the lead on it or aspects of it, working with their partner to know more about care arrangements and share some of the load. One father, David, described in detail his daughter's transition from child to adult services and his experience of navigating ‘the system’. This predominantly involved responding to emails from professionals and taking the lead in professional meetings. He reflected on the challenges associated with getting his daughter's care package in place now that she was an adult and how this aspect of the role was less straightforward and had an impact on his own mental health:

The caring for C is quite, we say the easy bit. It's all the stuff that goes with it…It is a constant, I'm going to say fight because it is a constant fight because it seems to have got even harder now she's turned 18… It's mentally draining and then like I say, you become emotional and your mental health suffers from all of this. (David)

Fathers also talked about the challenges associated with assessment processes, such as getting an Education and Health Care Plan (ECHP) for their child and the right educational provision in place and driving disputes. One father recalled his role in getting his son's EHCP:

I sort of drove getting his EHC in place…I did all the paperwork for that because we moved to [new location] just after he got diagnosed… there was a lot of work… (Peter)

#### 3.3.2 Subordinate Theme: Developing Their Own Knowledge

Obtaining first‐hand knowledge and information about their child's disability was important to fathers, particularly those with autistic children. Not knowing about autism in the early days when their child received their diagnosis negatively impacted their own mental health. Obtaining knowledge was therefore important for their own well‐being. Fathers described how they had been proactive in learning about autism. One father talked about the value of attending an autism course:

A course came up with—for autism that [my partner] had been and done, that actually I had the opportunity to go and do and spend some time kind of thinking through a lot of the stuff and how I interact with O and how I can finally make better connections with him. (Sean)

### 3.4 Superordinate Theme: Supporting Their Wider Family

#### 3.4.1 Subordinate Theme: Supporting Their Partner

Throughout the interviews, many fathers described how they supported their partner. They often recognised the emotional toll that caregiving had on their partner and supported them by providing comfort and, where possible, taking on more responsibility for caregiving. One father talked about how they were approaching their son's care and education as a team:

[My partner] doesn't always feel overwhelmed by school and the need to be managing that whole thing on her own. And I think it's that—it's that moving away from her feeling like she's doing it alone… (Sean)

#### 3.4.2 Subordinate Theme: Protecting Other Children

In families with multiple children, fathers talked about their role as a protector. They were often worried that their other children were not given as much attention or may be expected to take on responsibilities that were not age‐appropriate. Fathers were mindful of this and made a concerted effort to support their other children:

We want to make sure that he's not neglected at all because so much focus and attention has gone on to D that we—you know, she feels quite guilty and, you know, therefore puts in the effort and time with our son really to make sure that he's getting up to speed and he's getting what he needs as well, that he's not getting forgotten about. (Ranveer)

#### 3.4.3 Subordinate Theme: Providing for Their Family

A key role for fathers was being a provider for their family. All fathers talked about needing to work while trying to also be around as much as possible for their family, which often put additional strain on them. One father talked about trying to balance work with caring for his son:

Trying to do thirty‐seven and a half hours a week and be a carer and be this and be that, no it ain't easy, my bosses were not very sympathetic. (Richard)

Worries about the future also drove fathers to focus more on work. One father talked about how he was so worried at the beginning about the kinds of support that his son would need, he thought the best thing to do for his family was to work as hard as possible to be able to provide financially. His perspective on this later shifted after he suffered significant mental health difficulties. He spoke about his decision to engage more in family life:

When I took the step back, I just really just had a complete reset and I just sort of re‐thought everything. And I started going to all the sessions with A at school. And I just refocused everything. So, okay, rather than me working my butt off to sort of like provide, why don't I work my butt off to be here and be relevant and engage with everybody that's around here? (Sidney).

### 3.5 RQ2: Factors Impacting Fathers' Involvement, Roles and Responsibilities

The factors that emerged from the interviews were organised into three non‐hierarchical groups: individual, interpersonal and contextual (see Table 3).

**TABLE 3:** Factors influencing the involvement, roles and responsibilities of fathers of children with Intellectual and Developmental Disabilities.

Superordinate themes	Individual	Interpersonal	Contextual
Subordinate themes	Child characteristics	Communication with partner and child's mother	Flexible employment
	Father mental health	Opportunities to develop knowledge	Fathers in ‘female spaces’

### 3.6 Superordinate Theme: Individual Factors

#### 3.6.1 Subordinate Theme: Child Characteristics

Fathers reflected on how the additional needs of their child meant that their role looked different from that of fathers parenting a typically developing child. They talked about the intensity of caring over their child's lifetime, something which is different for the ‘average’ father. Fathers of adults described their continued role in providing personal care for their child and were more likely to refer to themselves as carers. One father, Martin, had given up work to be a formal carer for his teenage stepson to ensure that he could provide him with full and consistent care. Another, David, talked about how his 18‐year‐old daughter's care needs were ‘intense’ and required him to be a carer as well as be her parent. One father talked about working and therefore not being formally ‘classed as a carer’, but did perceive himself to be a carer because of the role he plays in his adult daughter's care:

Well, I am a carer…I have to live my life not as I would have done. So, my life was affected by it because I have to care for her so I don't know what classes you, makes you a carer. I'm not there as a main carer but I have to do it. (Greg)

#### 3.6.2 Subordinate Theme: Father Mental Health

Fathers at all life stages had experienced varying degrees of mental health struggles as they tried to come to terms with their child's diagnosis and navigate life with a disabled child. Some had experienced significant trauma in their child's early life. Some fathers sought formal support for this, but many had not, and felt isolated due to a lack of social network. The need to ‘stay strong’ for their partner was a recurring theme, with pressure to ‘make everything ok’ and ‘have all the answers’. Many fathers described throwing themselves into their work as this seemed like the best, most practical way of supporting their family, but then later felt guilty for doing this or reflected that it was most likely a coping or avoidance strategy:

I think initially, dads find that kind of throwing themselves into their work is the practical and the instrumental. It's the thing to do. And you know I've heard lots of you know similar stories in terms of that. It seems the only way at the start. I took an easier option out because that way, I didn't have to process a lot. (Sidney)

Some fathers described how at the beginning they grieved for the child and the life they thought they were going to have. For these fathers, it had an impact on their involvement in their early child's life, as they spent less time with their family to distract themselves:

I had big struggles, I'd never been so low I suppose, it was very challenging, I got to the stage where I didn't think I'd be able to sort of you know like move on, and at that time I realised that I wasn't fulfilling my duties properly, I was focusing on other things, often really negative things to try and distract myself from what was going on. (Jak)

Fathers described needing time to adjust and figure out what their role as a father would look like when raising a disabled child, but felt the pressure to stay strong for their partner:

I cried a lot, I went away and cried secretly, you know, to be away from…to me I had to be stoic, I had to be tough, because that's inbuilt in our society, it's never easy. You come home from doing a twelve‐hour day and have to be 100%, that's the way I always saw it. (Callum)

### 3.7 Superordinate Theme: Interpersonal Factors

#### 3.7.1 Subordinate Theme: Communication With Partner and Child's Mother

Communication between parents influenced the roles and responsibilities of fathers. In two‐parent households, many fathers described how they and their partner took on certain aspects of their child's care or routine (e.g., bedtime, admin related to child's care) to share the load. Some couples actively delegated tasks according to each other's strengths and preferences.

Doing the emails, doing Zoom meetings, and just, I try to take the pressure off my wife. It is easier for me to do the emails, it is easier for me to sort of like make sure that I am at that meeting… (David)

#### 3.7.2 Subordinate Theme: Opportunities to Develop Knowledge

Opportunities for fathers to learn more about their child's needs had a positive impact on father involvement. Those with more knowledge were more involved in many aspects of their child's care and demonstrated greater parenting self‐efficacy as they were more confident in identifying and supporting their child's needs. For some fathers, this meant that they felt as knowledgeable as their partner:

[My wife] is very knowledgeable, as we both are now. (David)

Fathers who received more first‐hand information about their child's care arrangements, by attending meetings and receiving regular communication, were more likely to be involved in the indirect care of their child. In some cases, this helped to reduce the pressure on the child's mother in relation to this aspect of caregiving.

### 3.8 Superordinate Theme: Contextual Factors

#### 3.8.1 Subordinate Theme: Flexible Employment

All fathers reflected on how work had affected the quantity and quality of their parental involvement. Some expressed guilt and felt that they were not able to do as much for their child (now or in the past), because of work responsibilities. Having flexibility in work often determined the amount and type of involvement fathers could have in their children's lives. Fathers also reported difficulty in trying to balance competing pressures, that is, the need to work and provide financially for their family, but also be present and productive in the home. Fathers expressed guilt when going out to work, but also discussed the negative impact that supporting their family had on their work identity and reputation as they felt that they came across as less dedicated or committed to their job:

Even though I'm self‐employed you know I feel torn if [daughter] is unwell and I've got a job, it's like ah shall I go or shall I stay? I end up not going and supporting [my partner] but obviously that doesn't bode well for my work. If I worked for someone then I would have got the sack ages ago! (David)

A change in employment meant that some fathers were able to attend more meetings about their child. One father reflected positively on how he was able to help his partner to prepare for and attend meetings and feel more involved in decision‐making:

I'm able to put much more input into it than I ever have done before in terms of helping through the documents and deciding how we're going to approach things and just being in every meeting. (Sean)

#### 3.8.2 Subordinate Theme: Fathers in ‘Female Spaces’

Interactions with professionals appeared to have some impact on fathers' involvement in meetings about their child. Fathers who were able to attend meetings recalled negative interactions they had experienced with professionals and services involved in their child's care, which they believed reflected the female‐dominated culture of services and expectations of how men should behave in those spaces. Fathers expressed annoyance that their involvement in their child's care was not always recognised or was dismissed, making them feel like the lower‐ranked parent. One father said that a professional had remarked that he was an ‘activist dad’ for being involved in his child's care. Fathers also said they were frustrated when actions were not followed through and felt that their questioning of decisions was perceived by female professionals as intimidating, which often resulted in their failing to attend or contribute to meetings. Two fathers recalled how a professional had called them ‘aggressive’ when raising concerns about their child's care:

[They will say] you're being aggressive. Well, actually, I'm not…I'm angry. There's a difference between being angry and aggressive and until we understand that, a lot of men, particularly you know a lot of men like me, we will rein it in 90%. (Richard)

Just because I'm a man and I'm a dad doesn't mean that I am aggressive, I'm passionate about what is going on. And it is not that I'm demanding this or demanding that, it is what [my daughter] needs. (David)

## 4 Discussion

This study has contributed to the limited research on the involvement, roles and responsibilities of fathers of children with Intellectual and Developmental Disabilities, especially on fathers of older children and adults. It describes what these fathers do with and for their children, and it has begun to understand factors that may be a barrier or facilitator to their involvement in their child's life.

### 4.1 Fathers' Involvement, Roles and Responsibilities

The findings revealed that the fathers in this study were very involved in the direct care of their child and performed parenting duties when not at work (Carpenter and Towers 2008). The involvement of fathers in their child's personal care at all life stages was also evident. In fathers of typically developing offspring, supporting their child with personal care is something which tends to tail off as the child reaches adolescence; however, for fathers (and mothers) of disabled adults, this aspect of care is likely to remain a key part of their role for a much longer time due to increased need, and something which they will require practical and emotional support for as they too grow older. In contrast to previous studies (Dunn et al. 2021; Thackeray and Eatough 2018), fathers of older children were more likely to say that they were a carer. While this may be because of differences in participant characteristics and generational attitudes towards what constitutes 'care', it may also reflect that special attention was paid to the roles and responsibilities of fathers in this study. It would be interesting to consider how aspects of caregiving may impact the identity formation of fathers over time.

Engaging in play and activities with their child was a significant and joyful part of parenting for the fathers, highlighting the importance of capturing positive parenting experiences (Hastings 2016). However, it was also evident that for many fathers, their parenting experience had been very different to what they had imagined and had taken some adjustment. These findings echo those of other studies where fathers have altered their expectations in response to their child's diagnosis (Davys et al. 2017; Rafferty et al. 2020), and indicate that fathers may require support with this aspect of coping.

It was evident that some fathers took on indirect care roles; however, these were mostly being performed by their partner and their child's mother. This finding is not surprising given that mothers of disabled children typically assume the majority of the administrative responsibilities related to their child (Dembosky et al. 2024; Nicholas et al. 2020). However, this study also showed that in some households, these responsibilities were not always solely within the mother's remit, with fathers—especially those of older children—liaising with services and driving assessments and disputes. It echoes other studies that have found that fathers can develop expertise about their child and have greater involvement in indirect care (Dunn et al. 2021).

The research also showed that many fathers were also supporting other family members, such as their partner and other children, in addition to their disabled child. These findings are in line with previous studies where fathers took on multiple roles within and outside of the family (Dunn et al. 2021; Schippers et al. 2020). For some fathers, it was clear that these multiple roles placed significant pressure on them, and thereforformal sources of support, which allow them to talk about these demands and perhaps even alleviate them through greater support for their child and partner, are warranted.

### 4.2 Factors Impacting Fathers' Involvement, Roles and Responsibilities

In line with the ecological model of Cabrera et al. (2014), the findings show that there are a range of factors at an individual, interpersonal and contextual level that can influence the involvement, roles and responsibilities of fathers. It was evident that individual factors, such as the age and needs of the child, impacted the fathers' parenting role. For those with older children with Intellectual Disabilities, direct and indirect care did not get any easier as the child grew older, despite some fathers growing in confidence, knowledge and skills. It suggests that fathers, like mothers, need help to navigate their role over their life course and are likely to directly benefit from respite care (Seymour et al. 2022).

It was evident that most of the fathers had experienced difficulties with their mental health following the diagnosis of their child's disability, commonly reported in the existing literature (Marsh et al. 2020). The impact of traditional gender norms was also evident with fathers demonstrating characteristics of hegemonic masculinity, such as stoicism (Connell 2005), in their attempts to hide their feelings and stay strong for their partner. These characteristics then appeared to have an impact on their role as fathers as they withdrew from their child and parenting responsibilities, which had a negative impact on their relationship with their child and partner. A hyper‐focus on work was often a coping strategy and meant that parents engaged in role specialisation (Hartley et al. 2014), whereby fathers took on the role of the ‘provider’ and mothers the ‘caregiver’. The findings demonstrate the potential impact of paternal mental health problems on parenting and family dynamics, which often gets overlooked. All fathers may have benefited from targeted mental health screening at the point of their child's diagnosis and at other pertinent times in their parenting journey (such as transition points), particularly as men are less likely than women to actively seek mental health support (NHS 2024).

In addition to individual factors, there were also interpersonal factors that influenced fathers' involvement, roles and responsibilities. Communication with their partner and child's mother ensured that aspects of the child's care and routine were delegated. Previous research has reported a positive relationship between fathers' cooperation with their partners and their involvement in the care of their disabled children (Brągiel and Kaniok 2014), and these findings point to attempts to negate role specialisation and have open conversations about their parental roles in the family. Obtaining knowledge and information was also found to be important for fathers' well‐being and involvement. Similar to previous research (Batchelor et al. 2021), fathers reported that they started their parenting journey without any prior knowledge or experience of disability and were more likely to cope and parent effectively once they learnt about their child's condition (Rafferty et al. 2020). It reinforces the importance of providing timely, first‐hand information to fathers, as it could have a significant impact on their parenting self‐efficacy.

Contextual factors such as flexible employment also had an impact on the type of involvement fathers had in their children's care. It was evident that a key part of their role as fathers was supporting their family financially, but they also experienced guilt and a desire to be more involved in daily caregiving and appointments for their child. Previous studies have found that raising a disabled child can put extra pressure on fathers to provide for their family and has an impact on their working life and employment choices (Carpenter and Towers 2008). Flexible working has the potential to negate this and positively impact a father's ability to be more involved in the care of their child; however, for many fathers, especially those in lower‐skilled jobs, this might not be an option unless they accept a drop in income. It is clear that fathers need more formal support to balance employment and care responsibilities.

The findings also highlighted the impact of gender stereotypes when fathers liaise with professionals working with their child. It is evident that fathers can feel like an outsider when attending meetings and that there are expectations which govern how they should behave in those ‘female‐dominated’ spaces. Other studies have reported that formal services are predominantly centred around the child and their mother (Seymour et al. 2022), which can lead to fathers feeling excluded. It highlights how societal expectations of men and fathers can act as a barrier to paternal engagement in formal services, and a need for professionals to critically review their provision and consider ways in which they can make their services more father‐friendly, perhaps through collaboration with male service‐users.

### 4.3 Limitations and Future Research

While the study captured the experiences of fathers of different ages, the data are still only cross‐sectional in nature, with fathers of older children providing retrospective accounts. A larger life‐span study which captures the involvement and role of fathers over time in the United Kingdom is needed. Asking about involvement can also be very subjective and may mean that fathers are more likely to present themselves favourably. Future research could look to triangulate with reports from other family members in order to capture a broader picture of family dynamics and relationships.

## 5 Conclusion

While the previous lack of research has been guided by the assumption that fathers of children with Intellectual and Developmental Disabilities are less involved in their children's lives, it is evident that the fathers included in this study were very engaged in the care of their children. Greater recognition of care demands and flexibility by employers would make a significant difference to the involvement of fathers in all aspects of care, along with targeted paternal mental health screening at the point of diagnosis and across the life course. Services working with families of individuals with Intellectual and Developmental Disabilities should explore ways to provide fathers with first‐hand information about their child's care and equip them with the knowledge they need to play an active role in their children's lives.

## Ethics Statement

The study received full ethical approval from the University of Warwick Humanities and Social Sciences Research Ethics Committee in November 2021 (HSSREC 37/21‐22).

## Conflicts of Interest

The author declares no conflicts of interest.

## Data Availability

Data from this research study are not available for sharing due to ethical approval requirements. Researchers interested in collaboration should contact Dr. Emma Langley.
